# Ultrasound-Assisted Extraction of Stilbenes from Grape Canes

**DOI:** 10.3390/molecules21060784

**Published:** 2016-06-16

**Authors:** Zulema Piñeiro, Almudena Marrufo-Curtido, Maria Jose Serrano, Miguel Palma

**Affiliations:** 1 IFAPA Rancho de la Merced, Carretera de Trebujena, Km. 2.2, Apdo. 589, Jerez de la Frontera 11471, Spain; almudena.marrucurti@mail.uca.es (A.M.-C.); mariajose.serrano@uca.es (M.J.S.); 2 Departamento de Química Analítica, IVAGRO, Universidad de Cadiz, Apdo. 40, Puerto Real 11510, Spain; miguel.palma@uca.es

**Keywords:** *trans*-resveratrol, stilbenes, ultrasound, extraction, HPLC, grape canes

## Abstract

An analytical ultrasound-assisted extraction (UAE) method has been optimized and validated for the rapid extraction of stilbenes from grape canes. The influence of sample pre-treatment (oven or freeze-drying) and several extraction variables (solvent, sample-solvent ratio and extraction time between others) on the extraction process were analyzed. The new method allowed the main stilbenes in grape canes to be extracted in just 10 min, with an extraction temperature of 75 °C and 60% ethanol in water as the extraction solvent. Validation of the extraction method was based on analytical properties. The resulting RSDs (*n* = 5) for interday/intraday precision were less than 10%. Furthermore, the method was successfully applied in the analysis of 20 different grape cane samples. The result showed that grape cane byproducts are potentially sources of bioactive compounds of interest for pharmaceutical and food industries.

## 1. Introduction

Stilbenes are non-flavonoid phenolic compounds expressed in many plant families [1]. Their occurrence in plant tissues is associated to plant resistance against fungal diseases such as *Botrytis cinerea*, or abiotic stress, such as UV irradiation. Hence, they are considered as phytoalexins [2]. These compounds have also attracted increasing attention because of the many benefits that they have on human health related to antioxidant, anticarcinogenic or cardioprotective [3,4,5] activities, among others.

Grape and red wine are among the major dietary sources of stilbenes [6], being present in both edible and non-edible plant tissues. The grape and wine industry is a valuable part of the economy in several regions around the world, with a total production area forecast to be 7,534,000 hectares in 2015 [7]. Most of the solid waste material generated from the viticultural and winemaking processes is usually composted or burned. Among these by-products, grape canes, which are usually discarded after the pruning season, can be considered as an unexploited source of stilbenes and other phenolics, as proposed by several authors [8,9].

The possible use of this material is gaining increased attention because of the promising applications in fields of cosmetic, food and pharmaceutical industries, and due to environmental concerns. In recent years, the Food and Agriculture Organization of the United Nations (FAO) predicted annual growth rates of 6%–8% for plant-based medical foods and phyto-pharmaceuticals. Pretreatment of the raw material and the extraction of valuable components are first steps in processing.

On the other hand, the analysis of plant stilbenes is usually performed by extracting the sample with aqueous organic solvents (mainly ethanol) and analyzing the extract by high performance liquid chromatography (HPLC), with detection by UV-vis, fluorescence or mass spectrometry [10,11,12]. Preparative chromatography is frequently used for isolation purposes [13,14]. However, these techniques often involve long extraction times and require several extraction steps.

Ultrasound-Assisted Extraction (UAE) appears to offer a solution to this problem as it is a technology that can accelerate mass transfer and enhance the extraction kinetics [15]. The ultrasound method is cheaper and easier to operate than other novel extraction techniques such as supercritical fluid extraction [16] or superheated liquid extraction [17]. The enhancement in the extraction efficiency of natural compounds from food matrices caused by ultrasound is attributed to the cavitation effect which enhances mass transport by disrupting the plant cell walls [18].

The aim of the study reported here was to develop a fast and simple method for the determination of stilbenes from grape canes, this process involved a UAE procedure followed by direct high-performance liquid chromatography analysis. Optimum extraction conditions based on UAE were identified and the final method was designed without including a solvent evaporation step, as recently described [19]. These authors macerated the grape canes overnight at room temperature as a suitable pretreatment and also included a solvent pre-concentration step before HPLC analysis. However, a faster method would allow the stilbenes to be determined in several different grape varieties in a short time. The work described here concerned the sample pre-treatment and development of a UAE-based method and its application to 20 different grape canes, many of which were analyzed for the first time (all assayed grape cane varieties except Sauvignon blanc, Carmenere, Zinfandel and Malbec cultivars), including compounds other than *trans*-resveratrol and *trans*-ε-viniferin, the main stilbenes identified in grape canes [19,20,21].

## 2. Results and Discussion

### 2.1. Study of Sample Pre-Treatment and Storage Conditions

Grape canes are generally only available in the pruning season (winter) and it was necessary to store and condition samples for them to be available during the development of the extraction method. Additionally, samples must be dried to guarantee microbiological stability and also the stability of some compounds. As a consequence, a suitable treatment method for sample conservation was required.

Several alternatives have been reported for the pre-treatment of grape canes and these are focused mainly on thermal drying [9,19] or freeze-drying [8,14]. Thermal drying, although the simplest method, can lead to losses by evaporation or decomposition, especially for thermo-labile compounds such as phenolics. This approach also requires long drying times, especially at room temperature (months), in comparison to oven-drying (from days to weeks). Freeze-drying is an interesting alternative because it is faster than thermal drying and the extent of some degradation reactions will be reduced, mainly because of the low temperatures used in the process.

Both drying methods will lead to an increase in concentration of the compounds because water is removed in both cases. The aim of the assay described here was to compare freeze-drying and thermal drying (the two most widely reported drying techniques) to evaluate the influence of the drying process on the recovery of stilbenes. Similar studies (including both freeze-drying and thermal-drying processes) have been reported for other solid samples and compounds: Keinanen and Julkunen-Tiitto [22] studied the effect of different drying methods on birch leaf phenolics and concluded that the highest concentrations of phenolics were obtained by freeze-drying; de Torres *et al.* [23] reported similar conclusions on the volatile and phenolic composition of grape skins and found that the freeze-drying method was less aggressive than thermal drying (60 °C) and the phenolic composition was better preserved than that in the thermally dried skins; Garcia-Perez *et al.* [24] reported that freeze-dried grape stalks presented the highest antioxidant concentration with the highest effective diffusivity and mass transfer coefficient and, furthermore, that the material was also more porous than thermally dried samples.

**Figure 1:**
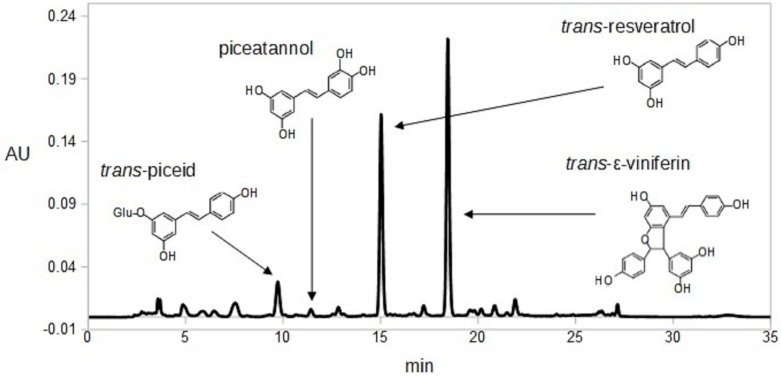
HPLC-DAD chromatogram corresponding to Malbec cultivar grape cane sample and structure of the studied compounds.

A total of 15 grape cane samples from several grape varieties (Sauvignon Blanc, Tempranillo, Malbec, Rome, Zinfandel, Tannat, Carmenere, Flame, Melissa, Red Globe, Moscatel Rosado, Victoria, Matilde, Crimson seedless and Tintilla de Rota) were cut into pieces, divided into two equal batches and the different drying procedures were carried out on the same scale. Samples were dried to constant weight using both techniques including an oven-dry at 40 °C for 15 days and a freeze-dried for 7 days. The average water loss for both procedures was 50% weight in which no significant differences were found for loss of water (data not shown). A typical chromatogram of grape cane and the structure of the different molecules studied are shown on Figure 1.

The samples were milled and then extracted. Experiments were carried out with 0.8 g of dried sample, which was placed in a volumetric flask (100 mL) made up to 25 mL with 80% ethanol in water and the same extraction conditions were applied (15 min extraction time, 70% amplitude, cycle 0.7, and 7 mm diameter tip, 75 °C) according to Piñeiro *et al*. [25]. All of these assays were carried out in duplicate with protection from light (flasks were covered with aluminum foil) to prevent degradation and/or isomerization by light.

**Figure 2:**
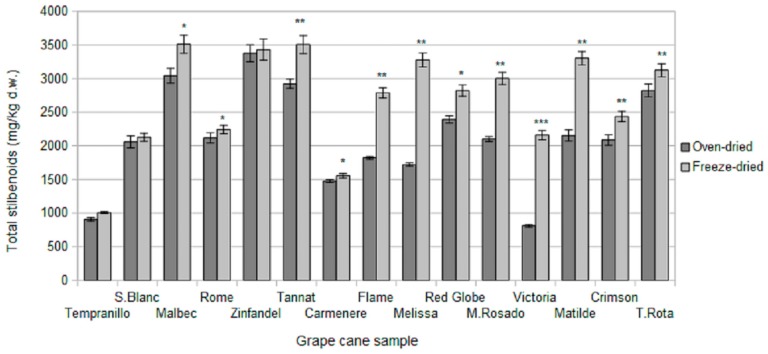
Total stilbenes in different assayed drying processes of grape canes. Bars with * symbol are significantly different at significance levels. * *p* < 0.05, ** *p* < 0.01 and *** *p* < 0.001.

The values for total stilbenes obtained in the extractions for different dried samples (Figure 2) were, in most cases, significantly higher (0.05 < *p* < 0.001) for freeze-dried samples than for oven-dried samples. There are only three cases (Tempranillo, Zinfandel and Sauvignon Blanc varieties) in which significant differences were not observed. In general terms, piceatannol was the only stilbenoid with higher contents in oven-dried samples (data not shown). Other compounds, such as *trans*-ε-viniferin, were present in similar quantities for both drying methods, whereas *trans-*resveratrol had significantly higher contents in the freeze-dried samples. Therefore, freeze-drying was the pre-treatment method used for grape cane samples when developing the UAE method. Similar results have previously been reported for other samples [22,23,24]. These results could be related to the mechanical effects on grape cane cells, as freeze-drying produce more intense cell wall degradation, thus allowing the solvent to penetrate deeper into the sample. Additionally, the use of high temperatures can promote degradation processes for some compounds.

Additional experiments were carried out with two grape cane samples in order to select the best storage conditions once the samples had been freeze-dried. Room temperature and freezer storage (−20 °C) were assessed after one month of storage. Significant differences were not observed for any of the quantified stilbenes (data not shown) and freeze-dried samples can therefore be stored at room temperature for easier handling without compound degradation.

### 2.2. Stability of Stilbenes

In order to evaluate the performance of different extraction conditions with accuracy, the stability of the stilbenes during the extraction was determined prior to method development. The stability study was performed using a stock standard solution of four commercially available stilbenes prepared in methanol.

One milliliter of stock solution was directly added to 25 mL of ethanol and submitted to the extraction conditions to check the stability of the stilbenes at 75 °C up to 35 min according to previous studies in our lab. Higher temperatures were not assayed because of the solvent will be evaporated after some minutes. Longer times were not assayed because of long extraction methods are not interesting for regular applications. Final composition in the resulting extract was compared to the starting composition of the stock standard dilution. The average recovery of all of the compounds in the sample was over 90%, e.g., piceatannol (96.7% ± 2.9%), *trans-*resveratrol (96.0% ± 0.4%) and *trans*-ε-viniferin (97.6% ± 1.0%). A temperature of 75 °C was therefore selected as the maximum temperature to be used when developing the UAE method, as in the extraction of real samples the use of a higher the temperature would lead to a faster extraction process.

### 2.3. Solvent Selection

Initial extractions were performed to determine the best solvent choice and these were carried out using a 1:30 sample-solvent ratio (0.83 grams of grape cane sample in 25 mL of each solvent) for 15 min according to previous studies. Palomino Fino 2011 samples were used throughout all of the method optimization assays. The assayed solvents, selected according to literature data [12,19,26,27,28], were as follows: ethanol, 80% ethanol in water (*v*/*v*), 80% methanol in water (*v*/*v*), 60% ethanol in water and 60% acetone in water. Selected solvents covered a wide range of boiling points (from 56 for acetone to 78 °C for ethanol) and three extraction temperatures were therefore assessed (25, 65 and 75 °C) to avoid artifacts related to solvent loss. The total stilbenes extracted with the different solvents and temperatures are shown in Table 1.

At 25 °C, the solvent that extracted the highest amount of stilbenes was 60% acetone in water, although significant differences were not observed with other solvents except for 100% ethanol, which extracted the lowest amount of stilbenes. Indeed, the use of 100% ethanol gave the lowest amounts of each stilbene at most of the temperatures assayed (except for 75 °C).

At 65 °C (a sufficiently high temperature to avoid total evaporation of each assayed solvent during the extraction time), the best solvent was 60% ethanol in water, the use of which led to the highest amounts of *trans*-ε-viniferin along with total stilbenes. In contrast, the methanol/water mixture was the solvent producing the lowest recoveries.

At 75 °C (the maximum extraction temperature used to develop the UAE method on the basis of stability results) the acetone/water mixture evaporated rapidly and it was therefore impossible to assess this solvent system. Ethanol/water mixtures were the most efficient solvents for all compounds and there was no significant difference between 60% and 80% ethanol for the extraction of stilbenes. It can be seen from the results that the extraction of samples at 75 °C led to a considerable increase in the stilbene content when compared to 25 °C: the total stilbenes concentration in the ethanol/water mixtures extracts increased to 89% (1362.9 *vs.* 721.9 mg·kg^−1^ dry weight) and 81% (1365.8 *vs.* 753.2 mg·kg^−1^ dry weight) achieved with 60% and 80% ethanol in water, respectively, in comparison to 35% (732.4 *vs.* 540.7 mg·kg^−1^ dry weight) in the ethanol extract. Therefore, it can be concluded that the use of ethanol/water mixtures at temperatures close to its boiling point is a better system for ultrasound transmission, thus allowing the ultrasound energy to reach the sample in a more effective way.

The use of 60% ethanol in water has several advantages over the use of 80% and these include environmental compatibility, lower toxicity and lower cost. These factors suggested the use of this solvent in the extraction method being developed. Thus 60% ethanol/water mixture was employed as the extraction solvent for subsequent optimization of the extraction conditions.

### 2.4. Sample-Solvent Ratio

A series of extractions using different sample-solvent ratios (1:20 to 1:50) were carried out at 75 °C for 15 min in order to evaluate the effect of the ratio on the extraction efficiency of stilbenes from grape canes. The amounts of the main compounds are presented in Table 2. In terms of total stilbenes the highest extraction efficiency was achieved using ratios between 1:40 and 1:50. As can be seen, the total amount of stilbenes extracted increased on increasing the sample–solvent ratio from 1:20 to 1:40. Significant differences were not observed between the ratios 1:40, 1:45 and 1:50. Based on these observations the sample–solvent ratio 1:40 was the most appropriate since it maximized the extraction efficiency while keeping the solvent volume as small as possible. Furthermore, the lower the solvent volume the shorter the time required to reach the extraction temperature. This ratio was therefore used for the subsequent optimization of the extraction conditions.

### 2.5. Extraction Time

With regard to the determination of the time required for the extraction, the recovery of the studied compounds was determined on applying extraction times from 5 to 35 min. The results are shown in Table 2. Significant differences were found for *trans*-ε-viniferin and total stilbene content on comparing times of 5 and 10 min, but this was not the case for *trans*-resveratrol. It can be seen from the results that the majority of the total stilbenes present in the sample were extracted within 5 min (approximately 92%) and an increase in the extraction time from 5 to 10 min led to an increase of 7.6 of the total amount of *trans*-ε-viniferin extracted. The use of longer extraction times (20–35 min) led to the same levels of recovery as 10 min, indicating that the quantitative extraction of stilbenes from the sample had occurred after this time. Therefore an extraction time of 10 min was employed.

In an effort to verify recovery, the number of extraction steps needed for total recovery was investigated. Thus, two successive extraction steps were carried out with fresh solvent, including a step that involved rinsing the solid between the first and second extractions. After first extraction, the sample was filtered under vacuum and the extract was collected and filled up to 25 mL. The solid was rinsed with 5 mL of fresh solvent (collected separately) and dried for 5 min. The sample was subsequently re-extracted with fresh solvent by the same procedure to provide a third fraction. All fractions were analyzed separately. The recoveries of the total stilbenes in each fraction were 926.9 (extract), 10.7 (rinse) and 25.0 (re-extract) mg·kg^−1^ dry weight, respectively. Most compounds were almost totally recovered in the first fraction (data not shown); however, evidence for the presence of all of the stilbenes was also found in the rinsing fraction. Therefore, a rinsing step is required in order to obtain quantitative recoveries (higher than 95%) for all the assayed compounds.

### 2.6. Analytical Characteristics of the Method

The precision of the method was studied in intra- and interday assays for each compound. This variation was assessed intraday by analysing replicates of the same grape cane sample (Table 3). The final relative standard deviations (RSD) (*n* = 5) ranged from 4.4% for *trans*-ε-viniferin and 8.9% for piceatannol. For interday analyses the relative standard deviations (*n* = 5) ranged from 4.5% (*trans*-resveratrol) to 10.0% (*trans*-piceid).

The accuracy of the method was established by determining the recovery of stilbenes spiked into the sample, in triplicate, according to the proposed method. One milliliter of the previously used stilbene stock standard solution was added to the sample before applying the extraction conditions. The mean recoveries for the analyzed samples (data not shown) ranged from 93.3% to 102.1% (*n* = 3) for piceatannol, *trans*-resveratrol and *trans*-ε-viniferin.

### 2.7. Comparison of the Method with Reference Solid-Liquid Extraction

The recoveries obtained using the newly developed UAE method were compared with those obtained by triplicate, using a previously described solid-liquid extraction method [19], with acetone/water mixture (6:4) employed overnight at room temperature. Significant differences (*p* < 0.01) were only found for *trans*-resveratrol, e.g., the *trans*-resveratrol concentration in the UAE extract was 49.2 mg·kg^−1^ dry weight versus 21.6 mg·kg^−1^ dry weight achieved with the method of Lambert *et al.* [19]. Significant differences were not detected for the other analyzed compounds or for the total content. Additionally, it should be noted that the new method enables a reduction of greater than 90% in the analysis time when compared to the SLE (overnight) method, with the same recovery achieved as for the UAE method. In addition, UAE extracts can be directly injected into the HPLC system without the need for the additional solvent removal step required in the SLE procedure (acetone/water).

### 2.8. Determination of Stilbenes in Grape Cane Samples

The optimized procedure was successfully applied to the determination of stilbene levels in 20 grape cane samples and the results are shown in Table 4. Most of the grape cane varieties assayed had not been analyzed previously and only results for Sauvignon Blanc, Carmenere, Zinfandel and Malbec cultivars were found in the literature.

Among the red grape varieties, the Tintilla de Rota grape cane sample (an autochthonous Andalusian grape variety) contained the highest amount of total stilbenes and also the highest concentrations of *trans*-piceid, whereas Zinfandel had the maximum *trans*-ε-viniferin content (Table 4).

Regarding white grape cane samples, Melissa was the variety that had the highest contents of *trans*-resveratrol, *trans*-piceid and piceatannol.

All of the studied stilbenes were found in all of the analyzed red grape cane samples. The most abundant stilbene in all grape canes was *trans*-ε-viniferin, followed by *trans*-resveratrol and *trans*-piceid.

The abundance of stilbenes differed depending on the cultivar. Tintilla de Rota showed a higher *trans*-ε-viniferin content (1964.8 ± 24.8 mg·kg^−1^ dry weight) than *trans*-piceid (952.8 ± 23.7 mg·kg^−1^ dry weight) and *trans*-resveratrol (575.1 ± 16.5 mg·kg^−1^ dry weight). In the Rome cultivar instead the most abundant stilbene was *trans*-ε-viniferin (1494.1 ± 18.8 mg·kg^−1^ dry weight) but followed by *trans*-piceid (348.4 ± 8.7 mg·kg^−1^ dry weight), being both higher than piceatannol (111.6 ± 4.2 mg·kg^−1^ dry weight) and *trans-*resveratrol (107.1 ± 3.1 mg·kg^−1^ dry weight) content. Surprisingly, low differences were found for the Palomino fino samples analyzed in different vintages, where the *trans*-resveratrol and *trans*-ε-viniferin contents remained quite similar. It is well known that stilbene content is influenced not only by cultivar, but also by climatic and cultural conditions in specific vintages.

The *trans*-ε-viniferin concentrations measured in our samples were in the range described by other authors for different varieties such us Pinot Noir: 433–1500 mg·kg^−1^ dry weight [26,28], Gewürztraminer, Cabernet Sauvignon, Cinsault, Moscatel Alejandría: 266–824 mg·kg^−1^ dry weight [24], several Estonian varieties (Hasaine Sladki, Zilga, Yubilei Novgoroda): 700–1700 mg·kg^−1^ dry weight [26], other major *Vitis vinifera* cultivars (such as Chardonnay, Sauvignon Blanc, Semillon, Syrah or Merlot): 967–3737 mg·kg^−1^ dry weight [19], or some wild-type species: 728–5739 mg·kg^−1^ dry weight [21].

With regard to *trans*-resveratrol content, the results reported in this paper are consistent with those described in the literature: 190–1526 mg·kg^−1^ dry weight [23], 100–3200 mg·kg^−1^ dry weight [26] and 383–6533 mg·kg^−1^ dry weight [20] in different major *Vitis vinifera* species, Estonian cultivars or several grape varieties grown in Chile, respectively. These concentrations were markedly higher than those reported recently by Houillé *et al.* [29] in different *Vitis vinifera* varieties, with values between 41 and 158 mg·kg^−1^ dry weight.

The high variability in *trans*-resveratrol content between the same cultivars and different growing regions has also been reported previously, e.g., values of 723, 1526 and 3400 mg·kg^−1^ dry weight for the Pinot noir variety in Chilean, French or Canadian vineyards, respectively [20,21,28]; or 529.2 and 1085 mg·kg^−1^ dry weight for the Zinfandel variety in a Spanish (in this study) and Chilean vineyards [20]. Therefore, the higher values reported here could be explained in terms of cultivar practices and climatic conditions in Southern Spain.

Piceatannol concentrations were in the range reported by Vergara *et al.* [24] for several cultivars, but are somewhat lower than those reported by other authors [19,21].

In general terms, the average total stilbene contents found in red grape cane samples were higher than those in the white grape samples, a finding that is consistent with the results reported by Lambert *et al.* [19].

Our findings were not unexpected since a number of abiotic or biotic stress factors, such as UV radiation, heavy metal ions or infection by fungi, are known to affect stilbene biosynthesis during grapevine growth. The differences observed between stilbene contents in *Vitis* samples and genotype have been reported previously [19,29,30].

## 3. Materials and Methods

### 3.1. Chemicals and Reagents

Analytical grade methanol, acetic acid, acetone and ethanol were supplied by Panreac (Barcelona, Spain). *trans*-Resveratrol (3,5,4′-trihydroxy-*trans*-stilbene), *trans*-piceid (polydatin; *trans*-resveratrol-3-*O*-beta-d-glucopyranoside) and *trans*-ε-viniferin were purchased from Extrasynthese (Lyon, France). Piceatannol (3,3′,4,5′-tetrahydroxystilbene) were purchased from Sigma-Aldrich (Steinheim, Germany). Ultrapure water from a Milli-Q system (Millipore Corp., Bedford, MA, USA) was used in this research.

### 3.2. Plant Material

All of the grape cane samples used (corresponding to 19 varieties tested) were taken from grapevines grown under the same warm climate conditions, at the same location and harvested in the 2013 vintage at the IFAPA-Rancho de la Merced Centre, in Jerez de la Frontera (Cádiz, Spain).

Grape canes (five white cultivars: Palomino fino, Sauvignon Blanc, Melissa, Victoria, Matilde; fourteen red cultivars: Tempranillo, Tintilla de Rota, *Vitis sylvestris*-1, *Vitis sylvestris*-2, Jaen tinto, Rome, Zinfandel, Tannat, Carmenere, Malbec, Flame seedless, Crimson seedless, Red globe, Moscatel rosado) were collected in the pruning season, dried with paper and stored at 4 °C along 9 months until the drying process was carried out. Two alternative sample drying processes (freeze-drying and oven-drying at 40 °C) and subsequent storage conditions (freezer or room temperature) were assessed. The final protocol selected was as follows: each sample was cut into pieces and these were immediately freeze-dried until maximum water loss was reached and the weight remained constant (representing a loss ranging from 40% to 50% of the original weight). The samples were subsequently crushed in a MM400 ball mill (Retsch, Haan, Germany) and were stored at room temperature prior to extraction.

### 3.3. Extraction Process

#### 3.3.1. Ultrasound-Assisted Extraction (UAE)

The extraction of stilbene compounds from grape canes by the application of ultrasound was initially performed by following an extraction protocol previously developed in our lab for other samples. This protocol was studied further in order to optimize the extraction of grape cane stilbenes: different solvent systems (EtOH, MeOH or acetone/water mixtures) and temperatures were evaluated. The development of the UAE method was carried out with a high intensity probe ultrasound generation system (200 W and 24 kHz, model UP 200S, from dr. Hielscher GmbH, Teltow, Germany). The amplitude controller allowed the ultrasonic vibrations at the probe microtip to be set at any desired level in the range 10%–100% of the nominal power. The cycle controller allowed the duration of the application of ultrasound to be set, to a fraction of a second, in the range 0.1–1.0. A thermostatic water bath was used to control the temperature during the extraction. The resulting extracts were filtered under reduced pressure and finally filtered through a 0.22 μm filter (PDVF Teknokroma, Barcelona, Spain) and stored at −18 °C prior to analysis.

#### 3.3.2. Reference Solid-Liquid Extraction (SLE)

SLE according to Lambert *et al.* [19] was also checked to corroborate the effectiveness of the ultrasound-assisted extraction. The resulting extract was centrifuged at 4,000 rpm for 5 min in a Digicen 20-R centrifuge (Orto Alresa, Madrid, Spain). The supernatant was collected and the solvent was removed under vacuum at a temperature below 35 °C using a rotary evaporator (Heidolph VV2001 rotavapor, Heidolph Instruments GmbH & Co., Schwabach, Germany). Dry samples were rediluted in 2 mL methanol/water (1:1) (HPLC grade), filtered through a 0.22 μm filter (PDVF Teknokroma, Barcelona, Spain) and stored at −18 °C prior to analysis.

### 3.4. Liquid Chromatography System

Chromatographic analysis was carried out on a Waters (Milford, MA, USA) high-performance liquid chromatography system equipped with a model 1525 pump and a Waters 996 Photodiode Array Detector. Separations were performed on a Mediterranea Sea18 column (Teknokroma, Barcelona, Spain) (RP-18, 25 × 0.46 cm; 5 μm particle size) and a guard column of the same material, at 30 °C. The mobile phases consisted of a water/methanol/acetic acid mixture, solvent A 88:10:2 and solvent B 8:90:2, at a flow rate of 1 mL·min^−1^. The elution program involved gradient elution from 35% B for 3 min to reach 50% B at 10 min, 70% B at 20 min and 100% at 23 to 28 min [31]. Empower software was supplied by Waters. For identification and quantification purposes, standards of *trans*-resveratrol, *trans*-piceid, piceatannol and *trans*-ε-viniferin were used.

### 3.5. Statistical Software

Significant differences among each variable were assessed by analysis of variance (ANOVA) and Tukey′s least significative difference (LSD) test using Statistix software, version 8.0, (Tallahassee, FL, USA).

## 4. Conclusions

A rapid (10 min), simple, reliable and quantitative method has been developed for the ultrasound-assisted extraction of stilbenes from grape cane samples. After the evaluation of several extraction variables, it was concluded that the optimized extraction conditions were: 75 °C as the extraction temperature, 1:40 sample-solvent ratio, 60% ethanol in water as the extraction solvent and an extraction time of 10 min. The proposed method is suitable to determine stilbene levels in grape canes by direct injection of the extracts with an analysis time of around 10% of the total time required for previous methods. The method also has high reproducibility (>90%). This study showed that grape canes, usually discarded agricultural byproducts, represented an interesting potential as an easily accessible source for high-value bioactive stilbenes of great interest in food industry and pharmacology.

## Figures and Tables

**Table 1:** Effects of different solvents on the extraction of total stilbenes (*n* = 3).

Solvent	Temperature	Total Stilbenes (mg kg^−1^ Dry Weight)
EtOH	25 °C	540.7 ± 11.1 ^a^
80% EtOH in water		753.2 ± 9.2 ^b^
60% EtOH in water		721.9 ± 19.1 ^b^
80% MeOH in water		765.4 ± 16.5 ^b^
60% acetone in water		831.9 ± 9.2 ^b^
EtOH	65 °C	579.1 ± 43.2 ^a^
80% EtOH in water		633.4 ± 30.0 ^a^
60% EtOH in water		689.5 ± 25.0 ^a^
80% MeOH in water		551.4 ± 14.0 ^b^
60% acetone in water		684.5 ± 28.0 ^a^
EtOH	75 °C	732.4 ± 26.0 ^a^
80% EtOH in water		1365.8 ± 23.0 ^b^
60% EtOH in water		1362.9 ± 19.8 ^b^
80% MeOH in water		578.7 ± 11.2 ^c^
60% acetone in water		-

Results are means ± SD (*n* = 3). Values in each extraction temperature followed by different letters are significantly different at *p* < 0.01.

**Table 2:** Effects of different conditions on the extraction of total stilbenes (*n* = 3).

Condition	Total Stilbenes (mg·kg^−1^ Dry Weight)
Sample–Solvent Ratio	
1:20	450.4 ± 11.0 ^a^
1:25	459.7 ± 9.5 ^a^
1:30	556.4 ± 11.7 ^b^
1:35	677.9 ± 19.9 ^c^
1:40	1005.1 ± 38.4 ^d^
1:45	998.0 ± 33.2 ^d^
1:50	995.1 ± 31.8 ^d^
**Extraction Time (min)**	
5	932.1 ± 22.5 ^a^
10	1014.5 ± 36.2 ^b^
15	997.10 ± 29.2 ^b^
20	1008.0 ± 31.5 ^b^
25	999.9 ± 28.9 ^b^
30	1003.8 ± 37.5 ^b^
35	1002.1 ± 44.1 ^b^

Results are means ± SD (*n* = 3). Values in each assayed condition followed by different letters are significantly different at *p* < 0.01.

**Table 3:** Intra- and inter-day precision of analyzed stilbenes in Palomino fino 2011 grape cane samples.

Precision	*trans*-Piceid	Piceatannol	*trans*-Resveratrol	*trans*-ε-Viniferin
	Intra-Day (*n* = 5)			
Mean (mg·kg^–1^ dry weight)	49.2	17.8	35.3	690.4
RSD (%)	8.8	8.9	6.2	4.4
	Inter-Day (*n* = 5)			
Mean (mg·kg^–1^ dry weight)	49.8	18.4	34.2	725.8
RSD (%)	10.0	9.5	4.5	4.9

RSD: relative standard deviation.

**Table 4:** Stilbene concentrations in twenty grape cane samples (*n* = 3) from the 2013 pruning season.

Cultivar	Cane Sample	*trans*-Piceid ^a^	Piceatannol ^a^	*trans-*Resveratrol ^a^	*trans*-ε-Viniferin ^a^	Total Stilbenes ^a^
White	Palomino Fino-1 *	50.6 ± 1.7	18.9 ± 0.6	37.5 ± 2.6	692.0 ± 5.9	799.0 ± 28.3
	Sauvignon Blanc	44.6 ± 0.4	28.9 ± 0.6	85.8 ± 2.1	1921.6 ± 26.7	2080.9 ± 73.2
	Palomino fino-2	32.3 ± 2.0	12.4 ± 0.2	48.7 ± 5.6	679.0 ± 10.2	772.4 ± 31.3
	Melissa	856.5 ± 20.9	160.2 ± 6.0	1529.4 ± 43.8	620.1 ± 7.8	3166.2 ± 108.8
	Victoria	430.7 ± 10.7	53.1± 2.0	710.4 ± 20.3	860.5 ± 10.9	2054.7 ± 62.1
	Matilde	811.4 ± 19.7	89.1 ± 3.4	863.2 ± 24.7	1421.6 ± 17.9	3185.3 ± 98.6
	Average white	371.0 ± 9.2	60.4 ± 2.1	545.8 ± 16.5	1032.5 ± 13.2	2009.7 ± 76.3
Red	Tempranillo	77.8 ± 2.8	30.2 ± 0.1	45.6 ± 1.2	807.2 ± 11.1	960.8 ± 73.8
	Tintilla de Rota	952.8 ± 23.7	66.0 ± 2.7	575.1 ± 16.5	1964.8 ± 24.8	3558.7 ± 81.4
	*Vitissylvestris*-1	30.1 ± 0.3	14.6 ± 0.2	122.5 ± 1.9	797.8 ± 6.6	965.0 ± 64.5
	*Vitis sylvestris*-2	43.2 ± 2.5	21.0 ± 1.3	104.9 ± 10.4	1332.5 ± 26.5	1501.6 ± 86.2
	Jaen Tinto	56.0 ± 1.7	21.3 ± 2.8	174.8 ± 7.7	431.4 ± 10.7	683.5 ± 68.9
	Rome	348.4 ± 8.7	111.6 ± 4.2	107.1 ± 3.1	1494.1 ± 18.8	2061.2 ± 38.7
	Zinfandel	319.1 ± 7.6	76.0 ± 2.8	529.2 ± 15.1	2296.1 ± 29.0	3220.4 ± 20.5
	Tannat	502.9 ± 12.9	44.2 ± 1.7	469.2 ± 13.4	2253.1 ± 28.4	3269.4 ± 23.9
	Carmenere	181.6 ± 4.3	37.3 ± 1.4	242.8 ± 6.9	973.1 ± 12.3	1434.8 ± 38.9
	Malbec	353.2 ± 8.5	76.5 ± 2.5	664.9 ± 19.0	2253.9 ± 28.4	3348.5 ± 61.6
	Flame seedless	199.1 ± 4.9	90.6 ± 3.2	598.4 ± 17.1	1715.6 ± 21.6	2603.7 ± 70.4
	Crimson seedless	419.4 ± 10.3	30.9 ± 1.2	237.5 ± 6.8	1587.8 ± 20.0	2275.6 ± 86.7
	Red Globe	391.3 ± 9.5	40.4 ± 1.5	389.7 ± 11.2	1784.7 ± 22.5	2606.1 ± 51.3
	Moscatel rosado	747.7 ± 18.2	67.6 ± 2.6	966.8 ± 27.7	1051.2 ± 13.3	2833.3 ± 66.1
	Average red	267.2 ± 8.3	57.7 ± 2.0	467.3 ± 11.3	1712.2 ± 19.6	2237.3 ± 62.1

^a^ mg·kg^−1^ dry weight ± SD. * From the 2011 pruning season.
